# Conventional DCs from Male and Female Lupus-Prone B6.NZM Sle1/Sle2/Sle3 Mice Express an IFN Signature and Have a Higher Immunometabolism That Are Enhanced by Estrogen

**DOI:** 10.1155/2018/1601079

**Published:** 2018-04-15

**Authors:** Michael H. Lee, Marita Chakhtoura, Uma Sriram, Roberto Caricchio, Stefania Gallucci

**Affiliations:** ^1^Department of Microbiology and Immunology, Lab of Dendritic Cell Biology, Lewis Katz School of Medicine, Temple University, Philadelphia, PA 19140, USA; ^2^Department of Medicine, Division of Rheumatology, Lewis Katz School of Medicine, Temple University, Philadelphia, PA 19140, USA

## Abstract

Type I interferons (IFN) are pathogenic in systemic lupus erythematosus (SLE) and were proposed to control the immunometabolism of dendritic cells (DCs). We previously reported that DCs from female lupus-prone mice constitutively overexpress IFN-responsive genes resembling the IFN signature found in SLE patients. As SLE has higher incidence in women than men, more so in women of reproductive age, estrogens are suggested to affect lupus pathogenesis. We investigated the effects of sex and estrogens on the IFN signature in conventional GM-CSF-bone marrow-derived DCs (cDCs), from male and female Triple Congenic B6.NZM.*Sle1/Sle2/Sle3* (TCSle) lupus-prone mice or from wild-type C57BL/6 mice, generated with titrations of 17-beta-estradiol (E2). We found that cDCs from prediseased TCSle male mice express the IFN signature as female TCSle cDCs do. Estrogens are necessary but not sufficient to express this IFN signature, but high doses of E2 can compensate for other steroidal components. E2 stimulation, regardless of sex, modulates type I IFN-dependent and type I IFN-independent activation of cDCs in response to TLR stimulation. Finally, we found that TCSle cDCs from both sexes have elevated markers of immunometabolism and estrogens enhance the metabolic pathways in cDCs, suggesting a mechanistic link between estrogens, immunometabolism, and the IFN signature in lupus.

## 1. Introduction

Systemic lupus erythematosus (SLE) is an autoimmune disease of complex pathogenesis characterized by autoantibodies against nucleic acids, chromatin, and ribonucleoproteins [1–3] as well as elevated type I IFN [1, 4]. Microarray analysis of PBMCs from SLE patients discovered the increased expression of IFN-responsive genes that coined the IFN signature [5–7]. Genome-wide association studies and genetic studies of families with SLE patients support a genetic dysregulation of IFN-*α* [8–12]. We have previously found that immune cells from NZM2410-derived Triple Congenic B6. NZM.*Sle1/Sle2/Sle3* (TCSle) [13] lupus-prone mice express a similar IFN signature *in vivo*, suggesting that these mice are a good tool to study the role of type I IFNs in lupus [14].

DNA and RNA are major autoAgs in lupus that can stimulate immune cells through TLR7 and TLR9 [15]. As major producers of type I IFNs in response to TLR7 and TLR9 signaling, plasmacytoid dendritic cells (pDCs) have been a major focus of SLE research [16–18], although recently, cDCs have been also appreciated. cDCs from SLE patients showed a dysregulated expression of immunomodulatory factors such as BLyS, CD86 [19–22], and PD-L1 [23] and induced greater allogeneic T cell proliferation than their healthy counterparts [20]. In TCSle mice, the IFN signature and *Ifnβ* expression *in vivo* are high in cDCs but not in pDCs [14], and TCSle-cDCs induce greater proliferation and IgM secretion from B cells *in vitro* [24]. Furthermore, murine studies with B6.*Sle1* mice demonstrated that TLR7 overexpression on cDCs, but not on pDCs, is responsible for driving splenomegaly and end organ damage [25]. Finally, modular transcriptional repertoire analysis of cells from SLE patients revealed an equal importance of IFN-*β* and IFN-*α* in the IFN signature [26], further underlying the importance of cDCs in lupus.

These cDC abnormalities could be due to an abnormal environment, intrinsic defects, or both in SLE. Among the possible environmental factors suspected to affect DC activation, sex hormones are important candidates. Indeed, they have long been thought to play a role in the pathogenesis of SLE, as the female to male prevalence ratio ranges from 4.3 : 1 to 13.6 : 1 [27]. As the onset of SLE is more frequent in women of childbearing age [27], estrogens could be responsible for this bias. In addition, Fulvestrant, a selective estrogen receptor modulator, decreased SLE disease activity index in a small clinical trial [28]. Murine models confirm a role for estrogens in lupus: overactivation of estrogen receptor-*α* (ER*α*) exacerbates lupus disease [29]. Further studies have determined that upon TLR7 or TLR9 stimulation, secretion of IFN-*α* from pDCs [30] and IL-6 from cDCs [31] is facilitated by estrogen/ER*α*. Different murine models of lupus with ER*α* deficiency have prolonged survival and reduced disease in females but not in males [32]. Early ovariectomy of female NZM2410 mice also reduced disease severity and splenic cDC numbers [33]. These results agree with the observations that 17-*β*-estradiol (E2) promotes dendritic cell differentiation through IFN regulatory factor (IRF) 4 in GM-CSF-derived DCs [34]. Interestingly, an opposite effect of estrogen is seen on Flt3 ligand-derived cDCs and pDCs: E2 inhibited the survival of DCs in Flt3 ligand in a dose-dependent manner [35]. As GM-CSF is involved in inflammatory pathways while Flt3 ligand is involved in homeostasis [36, 37], E2 is hypothesized to promote DC survival in inflammatory conditions. These studies were mostly performed in female mice; it remains unclear however how estrogens affect cDC activation in male lupus-prone mice and whether male cDCs express the IFN signature. Moreover, the mechanisms mediating the effects of estrogens on cDCs are not completely understood.

Recent studies have suggested a role for an increased immunometabolism in SLE [38]. Indeed, the pathways involved in the production of cellular energy were more activated in T cells from SLE patients and lupus-prone mice, and the metabolic inhibitors metformin and 2-deoxyglucose reduced disease severity in murine lupus [39–41]. To our knowledge, no study has determined the metabolic activity of DCs in SLE. Studies of wild-type murine cDCs indicate that, upon TLR stimulation, they undergo a metabolic shift towards aerobic glycolysis with a decline in oxidative phosphorylation regulated by nitric oxide [42, 43]. This change in the way in which cDCs produce their energy is necessary for cDCs to fully activate. In addition, type I IFNs were involved in the metabolic reprogramming that occurs during activation of wild-type cDCs [44] and pDCs [45] as important autocrine mediators. As E2 differentially regulates cDC survival in homeostasis and inflammation and the metabolic shift occurs during inflammation [35–37], it is important to investigate the role of estrogens in cDC metabolic changes in lupus.

We therefore investigated the effects of hormones, specifically E2, on the IFN-dependent and IFN-independent activation of cDCs from female and male lupus-prone mice. Different hormone receptor polymorphisms have been reported to modify SLE disease severity [46] and may affect different murine strains as well. Therefore, we investigated the effects of E2 and sex differences on cDC activation using TCSle mice [13] compared to its wild-type strain C67BL/6 (B6) to minimize possible differences in estrogen receptor signaling.

Our results demonstrate that cDCs from male TCSle mice express the IFN signature similar to cDCs from female lupus mice [14]. In addition, male and female cDCs are equally dependent on the presence of estrogen to activate and express the IFN signature. We found that estrogen is necessary but not sufficient for cDC expression of this IFN signature, but high doses of supplemented E2 can compensate for other steroidal components normally present in the culture media. Estrogen enhances cDC activation through the IFN-dependent and IFN-independent pathways in both wild-type and lupus cDCs. Finally, we found that lupus cDCs have higher metabolism than wild-type cDCs in both sexes and estrogen enhances the metabolic pathways in cDCs, suggesting a mechanistic link between estrogen, immunometabolism, and the IFN signature in lupus.

## 2. Material and Methods

### 2.1. Mice

C57BL/6 (B6) and NZM2410-derived Triple Congenic B6.*Sle1.Sle2.Sle3* (TCSle) [13] mice (purchased from The Jackson Laboratory, Bar Harbor, Maine) were bred and maintained in our animal facility. Studies were performed in accordance with the guidelines of the Institutional Animal Care and Use Committees of Temple University, a member of the American Association for the Accreditation of Laboratory Animal Care-accredited facilities. Age-matched female and male B6 and TCSle mice were used between 8 and 12 weeks of age, an age at which TCSle mice do not yet develop antinuclear autoantibodies in our animal facility.

### 2.2. *In Vitro* Bone Marrow-Derived Conventional Dendritic Cell (cDC) Cultures

Bone marrow precursors were flushed from the tibias and femurs of mice with 25-gauge needle and syringe, into a single-cell suspension, and then seeded at 10^6^ cells/mL/well in 24-well plates (Corning Costar, Tewksbury, Massachusetts) in complete IMDM. IMDM containing phenol red (Corning) was supplemented with 10% FBS (Gemini Bio-Products, West Sacramento, California), 100 units/mL penicillin, 100 *μ*g/mL streptomycin, 50 *μ*g/mL gentamicin, 55 *μ*M 2-ME (Life Technologies, Grand Island, NY), and 2 millimolar L-glutamine (Corning) for standard condition complete IMDM. Phenol red-free IMDM (Life Technologies) was supplemented with 10% charcoal-treated FBS (HyClone, Logan Utah or Omega Scientific, Tarzana, CA), penicillin/streptomycin, gentamicin, 2-ME (Life Technologies), and L-glutamine (Corning) for hormone-depleted complete IMDM. All media were supplemented with supernatant of a hybridoma cell line secreting murine GM-CSF. 17-*β*-estradiol (E2 from Sigma-Aldrich) was reconstituted in ethanol and added to hormone-depleted complete IMDM at concentrations of 0.03 nanomolar (nM), 0.1 nM, or 50 nM E2 with a final concentration of 0.1% ethanol. E2-devoid conditions were also supplemented with 0.1% ethanol. Alternatively, Fulvestrant (1 *μ*M or 100 nM in DMSO) and Tamoxifen (10 nM or 100 nM in ethanol) (Sigma-Aldrich) were added to standard condition media. One mL of media per well was added on day 2, and 1 mL of media was replaced on day 5 and each subsequent day until stimulation of cells. Resting cDCs were stimulated on day 7 of culture with 10 *μ*g/mL CpG-B 1826 (IDT Biotechnologies, Coralville, IA) or 1 *μ*g/mL R848 (Risiquimod, InvivoGen, San Diego, CA). Supernatants and cDCs were harvested at 6 or 24 hours post stimulation. One cDC culture from female and male B6 and TCSle mice was compared in the same experiment.

### 2.3. Quantitative RT-PCR

Gene expression in cDCs was analyzed by quantitative real-time RT-PCR in technical triplicates using TaqMan probes, as previously described [14, 47]. Briefly, total RNA was extracted using Qiagen RNeasy Plus kit (Qiagen, Valencia, CA) or Zymo quick kit (Zymo Research), following the manufacturer's protocols. cDNA was synthesized using the cDNA archive reverse transcription kit (Life Technologies). TaqMan primers and probes for *Cxcl10*, *Irf7*, *Isg15*, *Mx1*, and *Pdk1* were purchased from Applied Biosystems. *Cyclophilin* was used as the reference gene for normalization. The cycle threshold (Ct) method of relative quantification of gene expression was used for these TaqMan PCRs (ΔΔCt), and the normalized Ct values (against cyclophilin) were calibrated against the control sample (untreated WT female cDCs) in each experiment.

### 2.4. Flow Cytometry

cDCs were harvested 24 h post stimulation, washed in cold PBS, incubated with rat anti-mouse CD16/CD32 (clone 2.4G2, BioLegend) mAb for 10 min to block Fc*γ*Rs, and then stained for 30 min on ice with allophycocyanin-conjugated hamster anti-mouse CD11c (N418, eBioscience), PE-Cyanin7-conjugated rat anti-mouse CD11b (M1/70, BD Bioscience), either PE-conjugated rat anti-mouse CD86 (GL1, BD Bioscience) or PE-conjugated rat anti-mouse CD115 (AFS98, BioLegend), FITC-conjugated hamster anti-mouse CD80 (16-10A1, eBioscience) or FITC-conjugated hamster anti-mouse CD40 (HM40–3, BD Biosciences), and FITC-conjugated rat anti-mouse MHCII (M5/114.15.2 eBioscience) or Biotin-conjugated rat anti-mouse MHCII (M5/114.15.2 eBioscience) followed by streptavidin PerCPCy5.5 (eBioscience) or Biotin-conjugated goat anti-mouse MERTK (polyclonal R&D Systems) followed by streptavidin FITC. In selected experiments, cDCs were stained with fixable viability dye (FVD) ef780 (Invitrogen) for 15 minutes in cold PBS and then washed before being incubated with the above antibodies. Cells were fixed with 1% formaldehyde before acquisition on a FACSCanto cytometer (BD Biosciences). FlowJo (FlowJo LLC, Ashland, Oregon) software was used for data analysis.

### 2.5. ELISA

ELISA analysis was performed following the manufacturer's protocols in technical duplicates. ELISA kits were used to measure the protein levels of murine TNF-*α*, IL-12p70, IL-6 (BD Biosciences), and CXCL10 (R&D Systems, Minneapolis, MN) in the supernatants of cDC cultures in medium alone or stimulated with TLR ligands. Absorbance was measured at 450 nm with a VERSA max microplate reader (Molecular Devices) with SoftMaxPro 6.5 software. Absorbance at 570 nanometers was measured and used as wavelength correction.

### 2.6. Griess Reaction

Nitrites, a proxy for nitric oxide, were measured in cDC culture supernatants by Griess reaction. Technical duplicates of supernatants and standards (sodium nitrite, Sigma-Aldrich) were titrated in a 96-well plate (Corning Costar). Griess reagent (Acros Organics, Geel, Belgium) was then added to each well at a 1 : 1 ratio and absorbance was measured at 550 nanometers with a VERSA max microplate reader (Molecular Devices) with SoftMaxPro 6.5 software.

### 2.7. Statistical Analysis

Mean and SE were calculated by averaging the results of three to six independent experiments performed with independent bone marrow cultures obtained from individual mouse for each experiment. Prism software (GraphPad, San Diego, CA) was used for statistical analysis with the two-way unpaired *t*-test for comparison between two groups and the two-way ANOVA with Tukey multiple comparison for multiple groups. *P* values *p* < 0.05 were considered significant. ^∗^, Δ, and O represent *p* < 0.05. ^∗∗^, ΔΔ, and OO represent *p* < 0.01. ^∗∗∗^, ΔΔΔ, and OOO represent *p* < 0.001.

## 3. Results

### 3.1. Conventional DCs from Young Lupus-Prone Male Mice Express the IFN Signature

**Figure 1:**
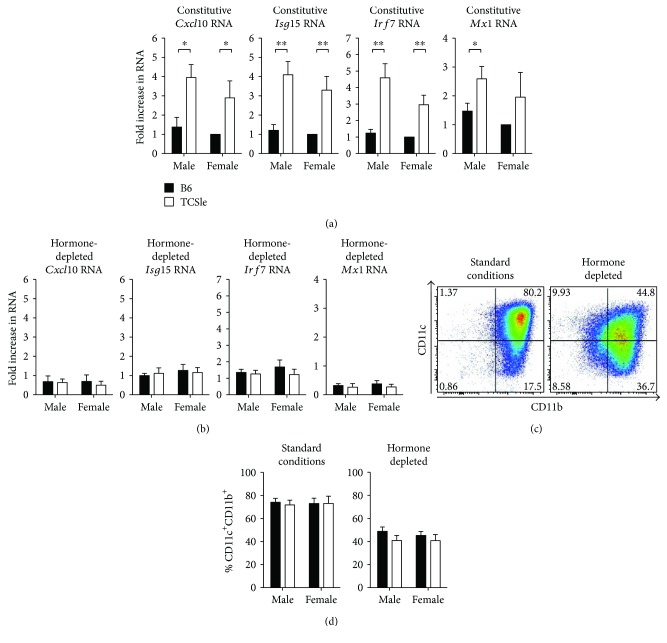
Conventional DCs from young lupus-prone male mice express the IFN signature. The IFN signature of TCSle cDCs is dependent on steroidal hormones. Bone marrow precursors from C57BL/6 (B6: black bars) or B6.NZM.Sle1.Sle2.Sle3 (TCSle: white bars) female and male mice were cultured with GM-CSF in standard phenol red/media conditions (a, c, d) or media lacking phenol red and void of steroids (charcoal-treated FBS: hormone depleted) (b, c, d). On day 7, cDCs were harvested and total RNA was isolated for qRT-PCR analysis (a, b). Genes were normalized to the housekeeping gene *cyclophilin*. Standard female B6 condition was set to 1. On day 7-8, cDCs were harvested and stained with antibodies against CD11c and CD11b and analyzed by flow cytometry (c, d). One representative plot of a B6 female in standard conditions (c, left) or hormone-depleted conditions (c, right). Unpaired *t*-test comparing male B6 to male TCSle or female B6 to female TCSle was used. Mean + SE values are from 6 independent experiments, using one mouse per strain per experiment. ^∗^ *p* < 0.05 and ^∗∗^ *p* < 0.01.

To test whether male lupus-prone cDCs express the IFN signature, we generated conventional dendritic cells (cDCs) from bone marrow precursors of female and male C57BL/6 (B6) and TCSle mice [13], in the presence of the DC growth factor GM-CSF, as previously described [14]. At day 7 under these standard conditions of culture (standard FBS & IMDM containing phenol red [14, 47]), we detected a higher baseline expression of interferon-stimulated gene (ISG) RNAs, namely, *Cxcl10*, *Isg15*, and *Irf7*, in female TCSle cDC compared to female B6 cDCs, confirming our previous findings [14] (Figure 1(a)). Although *Mx1* did not reach significance, there was a trend towards higher level of expression in female TCSle compared to female B6 cDCs. No significant difference was found between females and males of the same strain. Interestingly, male TCSle cDCs expressed significantly higher levels of *Cxcl10*, *Isg15*, *Irf7*, and *Mx1* transcripts compared to male B6 cDCs and similar levels to female TCSle cDCs. This indicates that male cDCs express a similar IFN signature to female cDCs from lupus-prone mice (Figure 1(a)).

### 3.2. The IFN Signature of TCSle cDCs Is Dependent on Steroidal Hormones

To determine the importance of sex hormones on the IFN signature, we cultured cDCs in hormone-depleted conditions. A well-accepted protocol to study the role of estrogen on cell functions uses a medium enriched with FBS that had been previously treated with charcoal to deplete all the steroidal hormones, including estrogens [35, 48]. We analyzed the differentiation of cDCs from bone marrow precursors grown in GM-CSF-enriched complete IMDM containing charcoal-treated FBS, and we used phenol red-free IMDM because phenol red has been suggested to signal through the estrogen receptor [49]. At day 7 of culture, we measured the IFN signature and found that it was abrogated in both female and male TCSle cDCs grown in hormone-depleted conditions (Figure 1(b)), in contrast to the results obtained in standard culture conditions of the same experiments (Figure 1(a)). To ensure that the lack of IFN signature was not due to B6 and TCSle cDCs developing at different rates in hormone-depleted conditions, we analyzed the expression of CD11c and CD11b as DC markers by flow cytometry (Figure 1(c); representative plot of B6 female). We found that the hormone-depleted conditions allowed the development of cDCs (~50% cDC after 7 days in culture) while standard culture conditions enhanced the frequency of cDCs (~75% cDC after 7 days). While there was a sizable reduction in the differentiation of DCs in hormone-depleted conditions compared to standard conditions, there was no significant difference between sexes and strains in cDC development within standard culture or hormone-depleted conditions (Figure 1(d)), indicating that the IFN signature in standard conditions was not due to a different ratio of B6 and TCSle cDC in culture. In summary, we used a protocol that allows the differentiation of bone marrow precursors into cDCs from both female and male B6 and TCSle mice in the absence of estrogens and other steroidal hormones. In these conditions, the lupus-prone cDCs lost the ability to express the IFN signature, while the baseline expression of ISGs by B6 cDCs of both sexes was not altered.

### 3.3. Estrogen Enhances the Development of cDCs of both Sexes of B6 and TCSle Mice and the IFN Signature in TCSle cDCs

**Figure 2:**
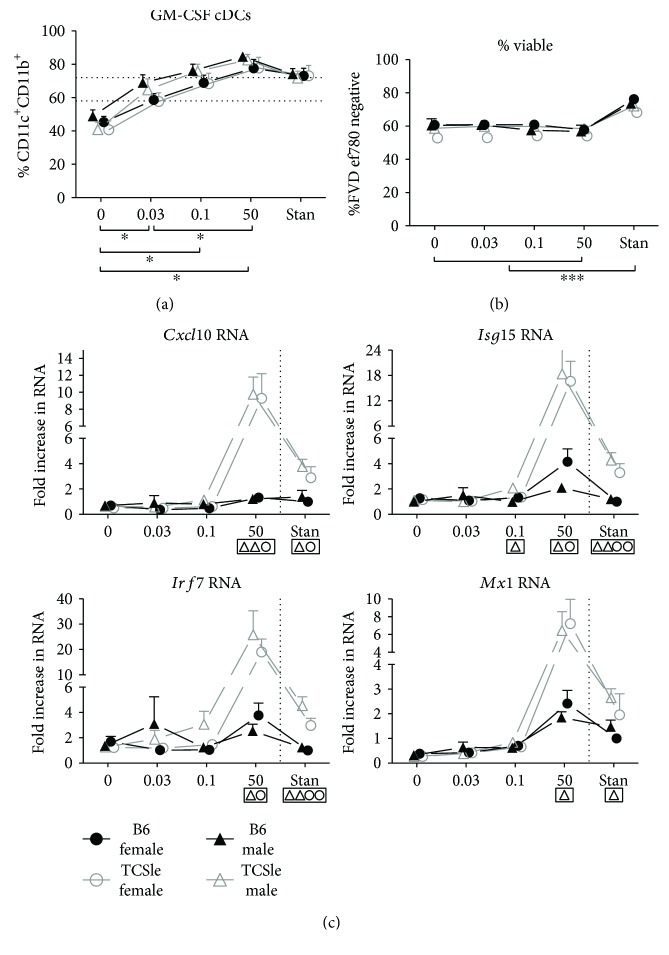
Estrogen enhances the development of cDCs of B6 and TCSle mice of both sexes and the constitutive IFN signature in TCSle cDCs. Bone marrow precursors from B6 (black closed symbols) or TCSle (gray open symbols) female (circle) and male (triangle) mice were cultured with GM-CSF in standard phenol red/media conditions or media depleted of phenol red and devoid of steroids (charcoal-treated FBS: 0 E2) and supplemented with 0.03 nM, 0.1 nM, or 50 nM 17-*β*-estradiol (E2). On day 7-8, cDCs were harvested and stained with (a) antibodies against CD11c, CD11b, and (b) fixable viability dye and analyzed by flow cytometry or (c) total RNA was analyzed by qRT-PCR analysis. ISGs were normalized to the housekeeping gene *cyclophilin*. Standard condition female B6 was set to 1 in each experiment. Mean + SE values are from 6 independent experiments, using one mouse of each strain and sex per experiment. Two-way ANOVA analysis with Tukey multiple comparisons was used to calculate the significance of the effects of E2 treatment within each group of mice represented by brackets below the graph. A black star indicates statistical significance in all the fours curves representing both sexes and strains of cDCs. Two-way ANOVA analysis with Tukey multiple comparisons was used to compare differences between B6 and TCSle, and results are shown in a box surrounding the symbol Δ for significance between B6 and TCSle males or the symbol O for significance between B6 and TCSle females. ^∗^, Δ, and O represent *p* < 0.05. ΔΔ, and OO represent *p* < 0.01. ^∗∗∗^ represent *p* < 0.001.

Estrogen signaling through IRF4 has been shown to enhance B6 DC differentiation *in vitro* [50]. We therefore determined whether estrogen enhances cDC differentiation and the constitutive expression of the IFN signature in cDCs from female and male TCSle mice. We generated cDCs in hormone-depleted medium supplemented with 17-*β*-estradiol (E2) at doses comparable to those present in the serum of male mice (0.03 nanomolar (nM)), in the serum of female mice in diestrus, or in a regular fetal bovine serum (0.1 nM) [51, 52]. We also tested a superphysiological dose that is comparable to what is present in the serum of pregnant women (50 nM) [53]. The lack of clear data in mice does not allow us to state that the latter concentration is equivalent to pregnant mice [54]. We found that E2 supplementation increased cDC differentiation compared to the lack of supplement, even at the lowest dose tested, which is equivalent to what is present in the serum of male mice (Figure 2(a)). We found an increase in the percentages of CD11c^+^CD11b^+^ cDCs, indicating a clear ability of E2 to enhance cDC differentiation, as previously reported [50]. Furthermore, in both sexes and strains, the dose present in the serum of female mice in diestrus (0.1 nM E2) was sufficient to yield the percentage of cDCs that we obtain under standard conditions (Figure 2(a)). To determine whether changes in differentiation were associated with differences in cell viability, we stained cDCs with a fixable viability dye and analyzed them by flow cytometry (Figure 2(b)). Although standard conditions resulted in significantly higher cell viability than the other conditions, E2 was not found to alter the percent of live cells compared to 0 E2. We observed no significant difference in cDC differentiation between female and male mice of both wild-type and lupus-prone strains, when the cDCs were generated in the same conditions and with the same concentrations of E2. This suggests that cDC differentiation is not affected by sex or the genetic make-up of the lupus-prone mice, but rather by the hormonal milieu in which cDCs grow.

As hormone-depleted conditions prevent the expression of the IFN signature in TCSle cDCs (Figure 1(b)), we determined whether E2 supplementation could rescue this phenotype. Surprisingly, neither 0.03 nM E2 nor 0.1 nM E2 supported an IFN signature in both female and male TCSle cDCs (Figure 2(c)). While the addition of 0.1 nM E2 and 50 nM E2 yielded a normal cDC differentiation (Figure 2(a)), only the 50 nM dose was capable of inducing higher levels of ISG expression in TCSle compared to B6 cDCs in both sexes (Figure 2(c)). Therefore, while high levels of E2 strongly induce the IFN signature in TCSle cDCs that we detected in standard conditions, levels of E2 similar to those present in male and in female mice outside of pregnancy cannot compensate for the absence of other steroids or lipids found in standard culture conditions.

**Figure 3 fig3:**
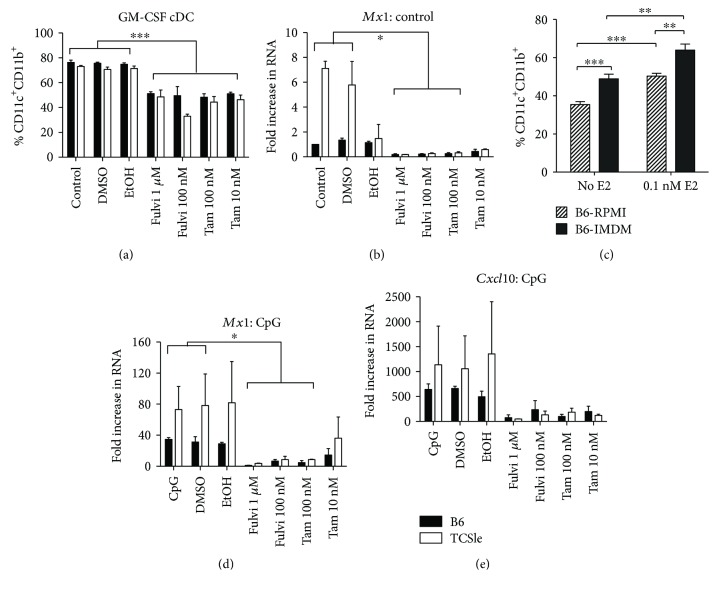
E2 inhibition reduces the development and IFN signature of TCSle cDCs. Bone marrow-derived cDCs from (a–e) B6 and (a, b, d, e) TCSle female mice were cultured with GM-CSF in standard phenol red IMDM (a–e) and RPMI (c) supplemented with Fulvestrant (Fulvi 1 *μ*M or 100 nM in DMSO) and Tamoxifen (Tam 10 nM or 100 nM in ethanol: EtOH). (a, b, d, e) or with 0 or 0.1 nM E2 (c). On day 7-8, cDCs were harvested and stained with antibodies against CD11c and CD11b and analyzed by flow cytometry. On day 7, cDCs were stimulated with CpG B 1826 (d, e). Six hours post stimulation, cDCs were harvested and total RNA was isolated for qRT-PCR analysis (b, d, e). *Mx1* and *Cxcl10* RNAs were normalized to the RNA of the housekeeping gene *cyclophilin*. Standard female B6 control condition was set to 1. Unpaired *t*-test comparing baseline cDC to SERM-treated groups was used. Mean ± SE values are from 2 independent experiments, using one mouse per strain per experiment (a–e). Brackets indicate significance between controls (including DMSO and EtOH) and SERM-treated samples. ^∗^ *p* < 0.05, ^∗∗^ *p* < 0.01, and ^∗∗∗^ *p* < 0.001.

In addition to removing steroids, charcoal treatment also removes other lipids from FBS and may affect the ability of cells to synthetize molecules and adjust their metabolism. As metabolites have been shown to alter DC growth and differentiation [55], we additionally tested cDC differentiation in standard conditions in the presence of selective estrogen receptor modulators/degraders (SERM/SERD) Tamoxifen and Fulvestrant. These compounds ablate E2 signaling while all other metabolites, which are removed by charcoal treatment, remained in the culture. cDC differentiation from female B6 and TCSle mice was significantly reduced by both Fulvestrant and Tamoxifen (Figure 3(a)). This reduction brought the percent of cDCs down to levels seen in hormone-depleted conditions (comparing Figure 3(a) to Figures 1(c) and 2(a)). In addition, Fulvestrant and Tamoxifen reduced the expression of the IFN signature, and we show the gene *Mx1* as representative (Figure 3(b)), supporting the role of estrogens in these functions.

In prior reports, hormone-depleted conditions yielded poorer B6 DC differentiation [56] than what we have seen in our experiments. Since those studies utilized the medium RPMI while our lab uses IMDM for GM-CSF cDC cultures, we generated cDCs in hormone-depleted conditions with phenol red-free RPMI or IMDM to determine if these two media, which contain different levels of amino acids and glucose, would yield a different percentage of cDCs. As we expected, IMDM promoted greater differentiation of cDCs than RPMI (Figure 3(c)). As with IMDM (Figure 2(a)), 0.1 nM E2 supplementation in hormone-depleted RPMI conditions was able to increase cDC differentiation (Figure 3(c)), as previously reported [56], although not at the same levels with IMDM supplemented with the same E2 concentration.

In summary, E2 supplementation equally enhances cDC differentiation in female and male B6 and TCSle cDCs, with the IMDM medium further promoting cDC differentiation. However, despite the ability of 0.03 nM E2 and 0.1 nM E2 to enhance cDC differentiation, only 50 nM E2 can rescue the IFN signature to the levels observed in standard conditions. As inhibition of the estrogen receptor by SERM/SERD blocks the IFN signature in TCSle cDCs generated in standard conditions, we conclude that estrogen is necessary but not sufficient for the expression of the IFN signature in lupus and only higher doses of E2 can compensate for other steroidal components present in FBS.

### 3.4. The IFN Signature of TCSle cDCs Is Not Due to a Differential Heterogeneity of the GM-CSF cDCs

**Figure 4 fig4:**
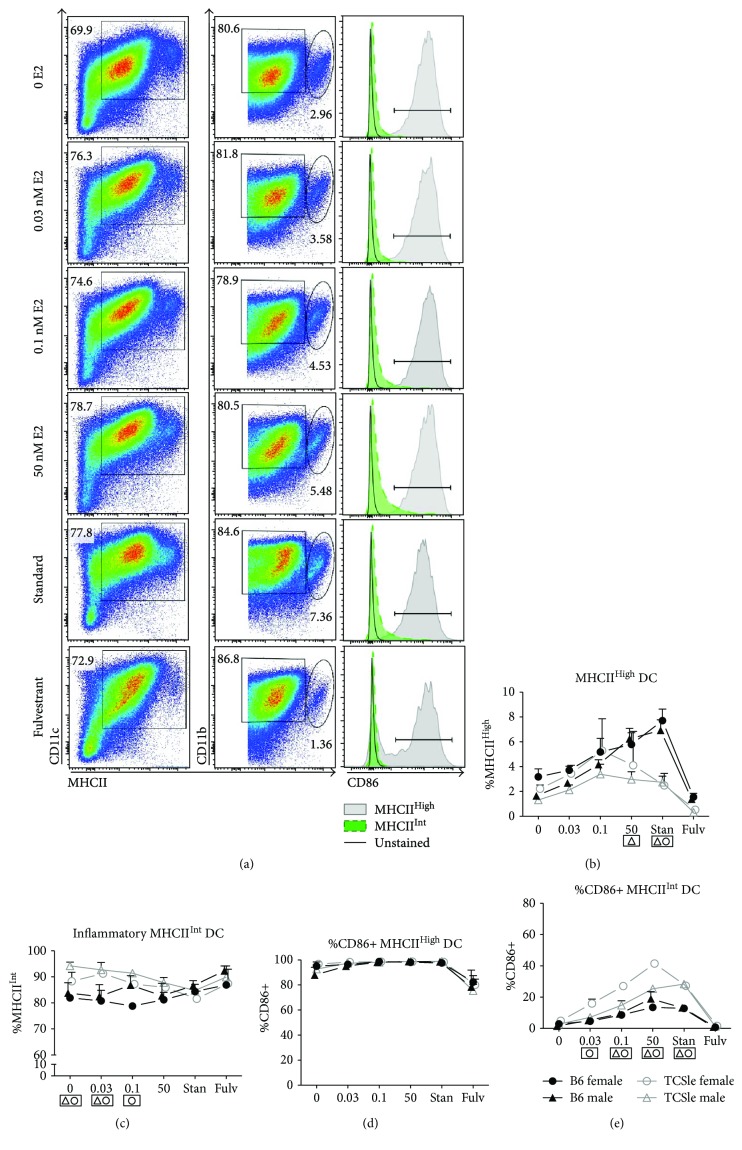
The IFN signature of TCSle cDCs is not due to a differential heterogeneity of the GM-CSF cDCs. Bone marrow precursors from B6 or TCSle female and male mice were cultured with GM-CSF in standard phenol red/media conditions or media depleted of phenol red and devoid of steroids (charcoal-treated FBS: 0 E2) and supplemented with 0.03 nM, 0.1 nM, or 50 nM 17-*β*-estradiol (E2). Standard conditions were also supplemented with 1 *μ*M Fulvestrant. On day 8, cDCs were harvested and stained with fixable viability dye and antibodies against CD11c, CD11b, MHCII, and CD86. Samples were gated on singlets, live cells, CD11c^+^ MHCII^+^ (a, left), CD11b + versus MHCII^High^ or MHCII^Int^ (a, center), and CD86 (a, right). Gray histograms represent the MHCII^High^ population, while green histograms represent the MHCII^Int^ population and the black line represents an unstained population. (a) One representative plot of a B6 female culture is shown. Graphs of %MHCII^High^ (b) or %MHCII^Int^ (c) from the CD11c^+^ gate from B6 (black closed symbols) or TCSle (gray open symbols) female (circle) and male (triangle) mice. Graphs of %CD86 positive on MHCII^High^ (d) or MHCII^Int^ (e) populations. Mean + SE values are from 3 biological replicates, using one mouse of each strain and sex per experiment. Two-way ANOVA analysis with Tukey multiple comparisons was used to compare differences between B6 and TCSle, and results are shown in a box surrounding the symbol Δ for significance between B6 and TCSle males or the symbol O for significance between B6 and TCSle females. Δ and O represent *p* < 0.05. ΔΔ and OO represent *p* < 0.01.

It has been previously shown that GM-CSF cDC cultures are composed of a heterogeneous population: CD11c^+^ MHCII^Int^, CD11b^High^ monocyte-derived inflammatory DCs, and CD11c^+^ MHCII^High^ CD11b^Int^ DCs. Since E2 titrations (Figure 2(a)) or SERM/SERDs (Figure 3(a)) could, respectively, increase or decrease the percent of CD11c^+^ CD11b^+^ cells, we determined if E2 affects the ratio between the reported CD11b^int^ and CD11b^high^ population of DCs. Representative gating of a B6 female is shown in Figure 4(a). Cells were first gated on CD11c^+^ MHCII^+^ (Figure 4(a), left) and then further divided between MHCII^High^ and MHCII^Int^ against CD11b (Figure 4(a), center). The MHCII^High^ population was high for CD86 (Figure 4(a), right (gray)) compared to the MHCII^Int^ (green) population. A similar trend was seen with CD40 (data not shown). Collectively, this suggested that the CD11c^+^ MHCII^High^ CD11b^Int^ cDCs are a spontaneously activated population as previously reported [57]. Interestingly, the MHCII^High^ population was not CD11b^Int^ as reported but higher in mean fluorescence intensity compared to the MHCII^Int^ population (Figure 4(a), center). We initially hypothesized that the TCSle cDCs may have an IFN signature because they were skewed towards a spontaneously activated MHCII^High^ DC population with E2 supplementation further enhancing this effect. However, in the conditions in which we saw an IFN signature (Figure 2(c): 50 nM E2 and standard conditions), there were less spontaneously activated MHCII^High^ TCSle DCs (Figure 4(b)) compared to B6 DCs and no difference between MHCII^Int^ TCSle DCs and B6 DCs (Figure 4(c)). Furthermore, when looking at an activation marker of these MHCII^High^ DCs, there were no differences between B6 and TCSle CD86 expression (Figure 4(d)). This spontaneous activation was modestly decreased with Fulvestrant. In contrast, the MHCII^Int^ DC population showed an E2-dependent, TCSle-specific, significant increase in CD86 (Figure 4(e)).

As these MHCII^Int^ CD11b^+^ CD11c^+^ have been referred to as more macrophage-like, we measured two surface markers: CD115, also known as M-CSF receptor, and MERTK. MHCII^Int^ DCs had an E2 dose-dependent increase in CD115 expression (Supplemental Figure 1A) which was neither strain nor sex specific (Supplemental Figure 1B). In contrast, we did not observe any MERTK staining in these experiments (data not shown), confirming our previous experience with GM-CSF cDC cultures done in the lab (unpublished data), although bone marrow-derived macrophage cultures generated in M-CSF-enriched medium regularly stained MERTK positive (unpublished data). In addition, the number of MHCII^High^ DCs in our culture ranged from 2–7% whereas Helft et al. reported around 30% [57]. We hypothesize that these differences in CD11b MFI, MER expression, and ratio of subsets may be attributed to culture media: while RPMI is a common media for GM-CSF DC cultures, we utilize IMDM, a richer medium, which yields a higher ratio of cDCs (Figure 3(c)) and may shift the culture towards a stronger dendritic cell phenotype.

In conclusion, while our GM-CSF DC cultures result in a heterogeneous population of inflammatory cDCs, the increase in IFN-responsive genes in the TCSle cDCs compared to B6 cDCs is not due to a change in cDC differentiation nor to an increase in the spontaneously activated MHCII^High^ cDCs, but rather to an augmented ability to express ISGs.

### 3.5. Estrogen Enhances the Upregulation of ISGs in Response to TLR Stimulation

**Figure 5 fig5:**
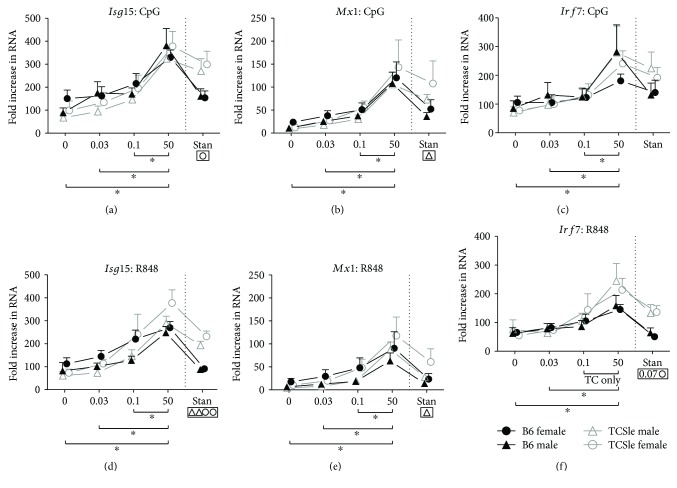
Estrogen enhances the upregulation of ISGs in response to TLR stimulation. B6 (black closed symbols) or TCSle (gray open symbols) female (circle) and male (triangle) mice were cultured with GM-CSF in standard phenol red/media conditions or media depleted of phenol red and void of steroids (charcoal-treated FBS: 0 E2) supplemented with 0.03 nM, 0.1 nM, or 50 nM E2. On day 7, cDCs were stimulated with CpG B 1826 (10 *μ*g/mL) (a–c) or R848 (1 *μ*g/mL) (d–f). Six hours post stimulation, cDCs were harvested for qRT-PCR analysis. ISGs were normalized to the housekeeping gene *cyclophilin*. Standard female B6 control condition without stimulation (not shown) was set to 1 in each experiment. Mean + SE values are from 6 independent experiments, using one mouse of each strain and sex per experiment. Two-way ANOVA analysis with Tukey multiple comparisons was used to calculate the significance of the effects of E2 treatment within each group of mice and the results are represented by brackets below the graph. A black star indicates statistical significance in all the fours curves representing both sexes and strains of cDCs. Two-way ANOVA analysis with Tukey multiple comparisons was used to compare differences between B6 and TCSle, and results are shown in a box surrounding the symbol Δ for significance between B6 and TCSle males or the symbol O for significance between B6 and TCSle females. ^∗^, Δ, and O represent *p* < 0.05. ΔΔ, and OO represent *p* < 0.01.

Nucleic acids are potent stimulators of the innate immunity and contribute to the IFN signature by stimulating DCs through TLR7/8 and TLR9 [14]. We therefore tested whether E2 enhanced the response to R848 and CpG, respective agonists of TLR7/8 and TLR9. We found that cDCs grown in hormone-depleted 0 nM E2 were able to respond to both CpG and R848 by upregulating the expression of the ISGs *Isg15*, *Mx1*, and *Irf7* (Figures 5(a)–5(f)). The highest concentration of 50 nM E2 further increased the expression of the ISGs *Isg15*, *Mx1*, and *Irf7* as compared to lower doses of E2 upon CpG stimulation (Figures 5(a)–5(c)) in both sexes and strains of mice. The highest E2 dose was similarly potent in enhancing *Isg15* and *Mx1* expression upon R848 stimulation (Figures 5(d) and 5(e)). Interestingly, while 50 nM E2 induced higher *Irf7* expression upon R848 stimulation, there was no statistical difference between 0.1 nM E2 and 50 nM E2 female and male B6 cDCs (Figure 5(f)). In contrast, TCSle cDCs were capable of further increasing *Irf7* expression with 50 nM E2. This may indicate an upper limit of *Irf7* expression in B6 cDCs that TCSle cDCs can surpass with higher doses of E2. Although we did not see consistent statistical significance because of variation between experiments, we observed a trend of higher expression of ISGs in TCSle cDC after CpG and R848 stimulation compared to B6 cDCs in standard conditions (Figures 5(a)–5(f): significance is shown in boxes below the graph). This suggests that both female and male TCSle cDCs increase their IFN signature upon TLR stimulation, as we have reported before in female TCSle cDCs [14]. In hormone-depleted E2-supplemented cDCs, the differences between TCSle and B6 cDCs of both sexes disappeared, suggesting that estrogen is necessary but not sufficient for the amplification of the IFN signature expression by TLR ligands in lupus. These conclusions are also supported by the findings that Fulvestrant and Tamoxifen, which inhibit E2 signaling, reduced the TLR-dependent upregulation of the ISGs *Mx1* and *Cxcl10* in cDCs generated in standard conditions (Figures 3(d) and 3(e)).

### 3.6. Estrogen Enhances the Upregulation of cDC Activation Markers in Response to TLR Stimulation

**Figure 6 fig6:**
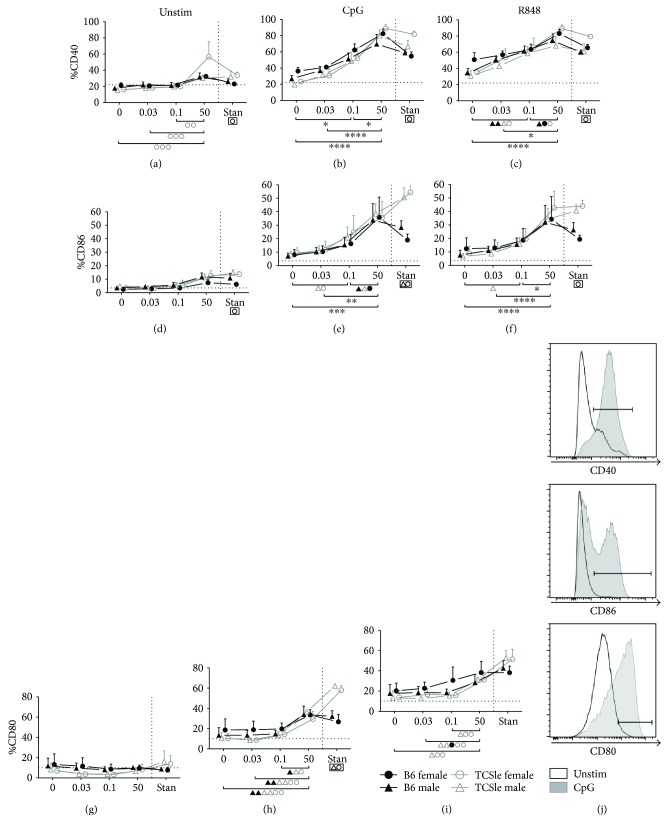
Estrogen enhances the upregulation of cDC activation markers in response to TLR stimulation. Bone marrow precursors from B6 (black closed symbols) or TCSle (gray open symbols) female (circle) and male (triangle) mice were cultured with GM-CSF in standard phenol red/media conditions or media depleted in phenol red and void of steroids (charcoal-treated FBS: 0 E2) supplemented with 0.03 nM, 0.1 nM, or 50 nM E2. On day 7, cDCs were stimulated with CpG (10 *μ*g/mL) (b, e, h) or R848 (1 *μ*g/mL) (c, f, i). cDCs were harvested 24 hours post stimulation, stained, and gated on singlets, live cells, and CD11c^+^ CD11b^+^ (gating shown in Figure 1(c)) before analyzing costimulatory molecules (a–i). Representative histogram plots of CD40, CD86, and CD80 on unstimulated (black line) or CpG-stimulated (gray histogram) female TCSle cDCs from standard conditions (j). Mean + SE values are from 3 (female cDCs) or 4 (male cDCs) independent experiments, using one mouse of each strain and sex per experiment. Two-way ANOVA analysis with Tukey multiple comparisons was used to calculate the significance of the effects of E2 treatment within each group of mice, represented by brackets below the graph. Black ^∗^ indicates significance within all 4 groups while symbols represent significance within a single group. Two-way ANOVA analysis with Tukey multiple comparisons was used to compare differences between B6 and TCSle, and results are shown in a box surrounding the symbol Δ for significance between B6 and TCSle males or the symbol O for significance between B6 and TCSle females. ^∗^, Δ, and O represent *p* < 0.05. ^∗∗^, ΔΔ, and OO represent *p* < 0.01. ^∗∗∗^ represents *p* < 0.001 and ^∗∗∗∗^ *p* < 0.0001.

As costimulatory molecules are dysregulated in SLE cDCs [19–21, 58] and can lead to abnormal T and B cell proliferation [20, 24], we next analyzed the effects of E2 on the expression of the surface activation markers CD40, CD86, and CD80 on cDCs, measured by flow cytometry (Figure 6). Constitutive expression of CD40 and CD86 was significantly elevated in TCSle female cDCs compared to B6 female cDCs in standard conditions (Figures 6(a) and 6(d): significance is shown in boxes below the graph), confirming the results from Figure 4(e). We did not find this difference in our previous work [58], although this may be due to the culture conditions in the past, when we used to grow cDCs in medium enriched with IL-4. Upon CpG and R848 stimulation, the percentage of CD40^+^ (Figures 6(b) and 6(c)), CD86^+^ (Figures 6(e) and 6(f)), and CD80^+^ (Figures 6(h) and 6(i)) cDCs significantly increased in standard culture conditions for all mice compared to unstimulated conditions (representative plot for CpG effects on TCSle is shown in Figure 6(j)). CpG and R848 stimulation induced higher CD40 expression in TCSle female but not in TCSle male cDCs as compared to B6 cDCs (Figures 6(b) and 6(c)). Upon CpG stimulation, CD86 and CD80 expression was significantly upregulated in male and female TCSle cDCs as compared to B6 cDCs in standard conditions, confirming that TCSle cDCs have a higher response to TLR7–9 compared to B6 cDCs as we have shown in gene expression (Figure 5).

CpG and R848 stimulation was able to increase CD40 and CD86 expression of cDCs cultured with 0.03 nM, 0.1 nM, and 50 nM E2. A horizontal dashed line provides visual aid to compare baseline to stimulated levels of CD40 (Figures 6(a)–6(c)) and CD86 (Figures 6(d)–6(f)). In contrast, only 50 nM E2 promoted CD80 activation in response to CpG and R848 (Figures 6(g)–6(i)). Increasing E2 doses further enhanced CD40 and CD86 expression (Figures 6(b), 6(c), 6(e), and 6(f): significance is represented by brackets below the graph).

Collectively, E2 supports the upregulation of costimulatory molecules CD40 and CD86 in response to TLR7 and 9 stimulation. The absence of any difference between females and males when cultured with the same concentration of E2 indicates that the E2-specific hormonal environment, not the sex of the cDCs, dictates activation levels. However, female TCSle cDCs show higher expression of costimulatory molecules than female B6 cDCs in standard conditions while male DCs did not show consistent strain differences. Therefore, the upregulation of costimulatory molecules in cDCs is controlled by E2 in a sex-independent manner and by additional factors in a sex-dependent manner.

### 3.7. Estrogen Enhances CXCL10 Chemokine Production Not Predicted by *Cxcl10* RNA

**Figure 7:**
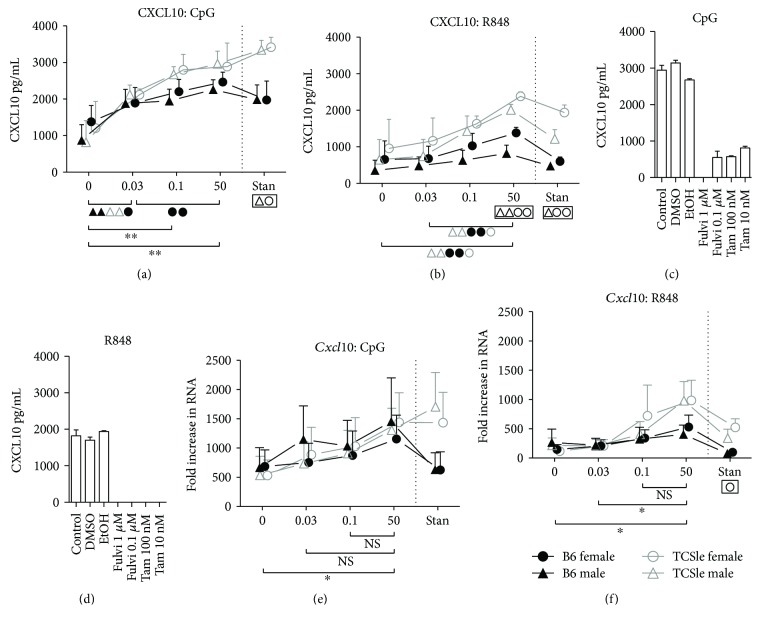
Estrogen enhances CXCL10 chemokine production not predicted by *Cxcl10* RNA. Bone marrow precursors from B6 (black closed symbols) or TCSle (gray open symbols) female (circle) and male (triangle) mice were cultured in standard conditions or hormone-depleted conditions supplemented with 0.03 nM, 0.1 nM, or 50 nM E2 (a, b, e, f). TCSle cDCs were cultured in standard conditions supplemented with Fulvestrant (Fulvi 1 *μ*M or 100 nM in DMSO) and Tamoxifen (Tam 10 nM or 100 nM in ethanol) (c, d). On day 7, cDCs were stimulated with CpG (10 *μ*g/mL) (a, c, e) or R848 (1 *μ*g/mL) (b, d, f). Supernatants were harvested 24 hours post stimulation and analyzed by ELISA for CXCL10 protein levels (a–d). Total RNA was isolated 6 hours post stimulation and analyzed by qRT-PCR (e, f). Mean + SE values are from 3 (female cDCs) or 4 (male cDCs) independent experiments or (c and d) 2 independent experiments using one mouse per strain per experiment (a, b, e, f). Two-way ANOVA analysis with Tukey multiple comparisons was used to calculate the significance of the effects of E2 treatment within each group of mice, represented by brackets below the graph. Black ^∗^ indicates significance within all 4 groups while symbols represent significance within a single group. Two-way ANOVA analysis with Tukey multiple comparisons was used to compare differences between B6 and TCSle, and results are shown in a box surrounding the symbol Δ for significance between B6 and TCSle males or the symbol O for significance between B6 and TCSle females. ^∗^, Δ, and O represent *p* < 0.05. ^∗∗^, ΔΔ, and OO represent *p* < 0.01.

We have previously reported that cDCs from TCSle female mice, grown in standard conditions, secrete significantly more CXCL10 chemokine than B6 cDCs upon CpG and R848 stimulation, as part of the IFN signature [14]. Therefore, we hypothesized that male TCSle cDCs can also secrete elevated levels of CXCL10 protein upon the same stimulations. Indeed, we found that upon CpG (Figure 7(a)) and R848 (Figure 7(b)) stimulation, CXCL10 production was significantly elevated in female and male TCSle cDCs as compared to their B6 cDC counterparts under standard conditions (significance is shown in boxes below each graph), further supporting the idea that male cDCs have an IFN signature that is amplified by TLR7–9 stimulation as female cDCs do.

Furthermore, CXCL10 production upon CpG stimulation was elevated in both female and male TCSle cDCs as compared to B6 DCs at 0.1 nM E2 and 50 nM E2, although it did not reach significance (Figure 7(a)). Moreover, CXCL10 production by TCSle cDCs was significantly higher than that by B6 cDCs in 50 nM E2 conditions upon R848 stimulation with a similar trend at 0.1 nM E2 (Figure 7(b)), suggesting that estrogen can enhance the amplification of the IFN signature induced by TLR7 and TLR9 stimulation.

While only higher concentrations of E2 promoted a difference between TCSle and B6 in CXCL10 production, even 0.03 nM E2 was sufficient to enhance CpG-induced production of CXCL10 compared to hormone-depleted conditions in both sexes and strains (Figure 7(a): significance is shown in brackets below each graph). Interestingly, putting aside the enhancing effects of E2, there was still significant production of CXCL10 in hormone-depleted conditions upon CpG stimulation (Figure 7(a)). In contrast, upon R848 stimulation, hormone-depleted conditions and 0.03 nM E2 were not capable of inducing as robust of a CXCL10 response (Figure 7(b)), indicating that E2 may affect CXCL10 production in response to CpG and R848 differently. To confirm this hypothesis, we cultured female TCSle cDCs in standard culture conditions with Tamoxifen and Fulvestrant to inhibit estrogen receptor signaling. In concordance with the response to CpG seen in hormone-depleted conditions and E2 supplemented, both high and low doses of Tamoxifen can reduce CXCL10 production but are unable to ablate the CXCL10 response to CpG stimulation (Figure 7(c)). Similarly, a low dose of Fulvestrant reduced CXCL10 production to similar levels seen in hormone-depleted conditions, while the higher dose of Fulvestrant prevented CpG-induced CXCL10 production (Figure 7(c)). We found that both low and high doses of Tamoxifen and Fulvestrant ablated the CXCL10 response to R848 (Figure 7(d)), suggesting that TLR7-induced CXCL10 is more estrogen dependent than TLR9-induced CXCL10.

We have shown in Figure 5 that only the highest dose of 50 nM of E2 could enhance the RNA expression of ISGs upon CpG and R848 and it was not sufficient for the expression of a significant difference between TCSle and B6 cDCs. In Figures 7(a) and 7(b) instead, we show that all 0.03 nM, 0.1 nM, and 50 nM could enhance the TLR9-induced production of ISG CXCL10 as protein and 50 nM allowed the TCSle cDCs to secrete significantly higher amounts of CXCL10 upon TLR-7 stimulation than B6 cDCs. To solve the contrast between the results presented in Figure 5 and Figure 7 [59], we determined if the regulation of CXCL10 production by E2 was detectable at the RNA level. Surprisingly, the ability of E2 to enhance CXCL10 production upon CpG stimulation was not mirrored in *Cxcl10* transcript levels (Figure 7(e)), as only 50 nM E2 supported elevated *Cxcl10* levels of expression as compared to hormone-depleted conditions. However, upon R848 stimulation, *Cxcl10* RNA levels more closely mirrored the production of CXCL10 protein (Figure 7(f)).

In summary, estrogen enhances the production of the chemokine CXCL10 in a dose-dependent manner, as we have observed with the upregulation of costimulatory molecules, in both strains and sexes of cDCs (Figure 6). E2 also strengthens the amplification of the IFN signature induced by TLR7 and TLR9 stimulation in lupus cDCs. Since we found different susceptibilities to estrogen in the production of CXCL10 protein levels versus *Cxcl10* transcripts upon CpG versus R848 stimulation and since the results of the RNA expression of CXCL10 were in agreement with the results of the gene expression of the other ISGs shown in Figure 5, we propose that the regulation of CXCL10, and possibly other ISGs, by E2 occurs at both the transcriptional and posttranscriptional levels.

### 3.8. E2 Enhances both IFN-Dependent and IFN-Independent Cytokine Production

**Figure 8:**
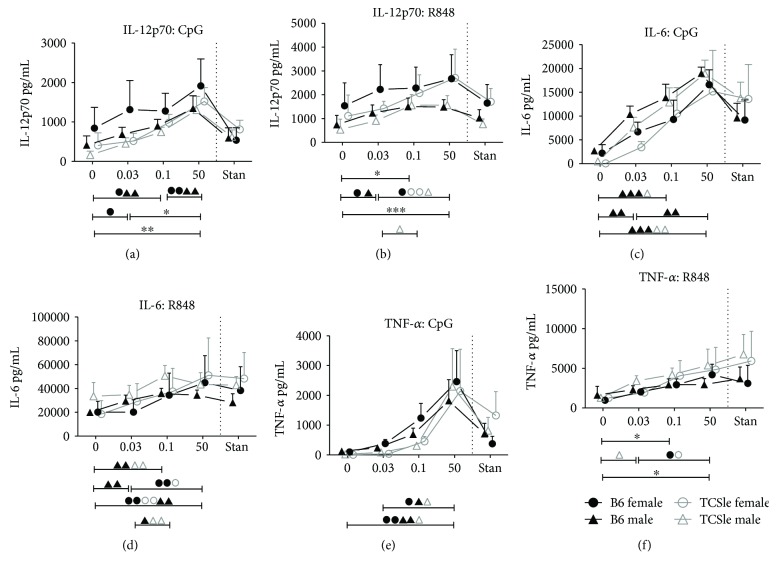
E2 enhances both IFN-dependent and IFN-independent cytokine production. Bone marrow precursors from B6 (black closed symbols) or TCSle (gray open symbols) female (circle) and male (triangle) mice were cultured with GM-CSF in standard phenol red/media conditions or media depleted in phenol red and void of steroids (charcoal-treated FBS: 0 E2) supplemented with 0.03 nM, 0.1 nM, or 50 nM E2. On day 7, cDCs were stimulated with CpG (10 *μ*g/mL) (a, c, e) or R848 (1 *μ*g/mL) (b, d, f). Supernatants were harvested and analyzed by ELISA 24 hours (IL-12p70 (a, b)) or 6 hours (IL-6 (c, d) and TNF-*α* (e, f)) post stimulation. Mean + SE values are from 3 (female cDCs) or 4 (male cDCs) independent experiments, using one mouse of each strain and sex per experiment. Two-way ANOVA analysis with Tukey multiple comparisons, used to compare differences between B6 and TCSle, did not reveal significance between the strains. Two-way ANOVA analysis with Tukey multiple comparisons was used to calculate the significance of the effects of E2 treatment within each group of mice, represented by brackets below the graph. Black ^∗^ indicates significance within all 4 groups while symbols represent significance within a single group. ^∗^, Δ, and O represent *p* < 0.05. ^∗∗^, ΔΔ, and OO represent *p* < 0.01. ^∗∗∗^ represents *p* < 0.001.

We have shown so far that E2 regulates the expression of ISGs (Figures 2 [Fig fig3] [Fig fig4] [Fig fig5] [Fig fig6]–7), costimulatory molecules (Figure 6), and the chemokine CXCL10 (Figure 7), equally in female and male cDCs, and contributes to the differences between TCSle and B6 cDCs. We have previously reported that ISGs, some costimulatory molecules, and CXCL10 are regulated in a type I IFN/STAT-2-dependent manner [47]. To assess whether E2 can also affect IFN-independent functions in TCSle cDCs, we measured the levels of IL-12p70, IL-6, and TNF-*α*, cytokines that we have previously shown to be IFN independent [47]. We analyzed the same supernatants in which we had measured CXCL10 (Figure 7). As with CXCL10, the secretion of IL-12p70 upon CpG (Figure 8(a)) or R848 (Figure 8(b)) stimulation was enhanced by 50 nM E2 (significance is shown in brackets below each graph) in both female and male B6 and TCSle cDC. In addition, 0.1 nM E2 significantly enhanced the IL-12p70 response to R848 but not CpG in all mice (Figure 8(b)). Although it did not reach significance for all mice, there was a trend towards 0.03 nM E2 supporting a stronger IL-12p70 response upon CpG and R848 stimulation compared to hormone-depleted conditions. In similar fashion to IL-12p70 and CXCL10, increasing E2 titration increased the secretion of IL-6 (Figures 8(c) and 8(d)) and TNF-*α* (Figures 8(e) and 8(f)) upon CpG and R848 stimulation, although it did not reach significance for all mice. In contrast to CXCL10, the secretion of IL-12p70, IL-6, and TNF-*α*, IFN-independent cytokines, was produced in equal amounts by TCSle and B6 cDCs (Figure 8), confirming our previous report [14], and did not show sex differences. Moreover, the enhancement of these cytokines by estrogen was similar in both strains and sexes.

In summary, E2 enhances the production of IFN-independent cytokines in both sexes in wild-type and lupus-prone strains. These results indicate that the enhancement by estrogen of the response of cDCs to TLR7 and TLR9 ligands involves both the IFN-dependent and IFN-independent pathways, suggesting that estrogen affects more than one mechanism, possibly upstream of the production of type I IFNs.

### 3.9. E2 TCSle cDCs Show a Higher Energy Metabolism

It was recently shown that T cells from TCSle female mice have an elevated metabolic state, with measures of increased glycolysis and oxidative phosphorylation, as compared to B6 T cells [41]. No data is available on the metabolic state of TCSle cDCs. To determine the effects of E2 on cDC metabolism, we chose two biomarkers of metabolic activation of the cDCs, Pdk1, and nitric oxide (NO).

**Figure 9:**
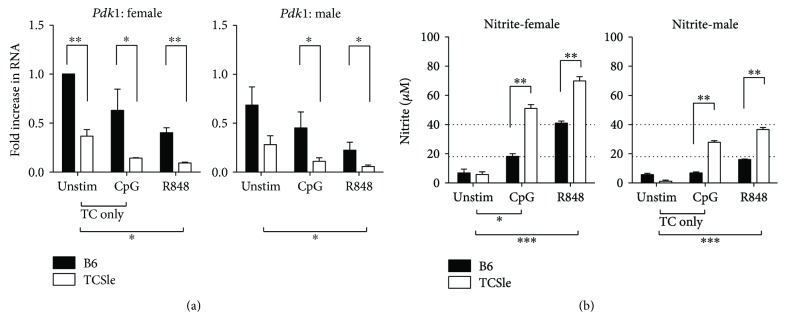
Female and male TCSle cDCs have a higher immunometabolism. Bone marrow-derived cDCs from B6 and TCSle female and male mice were cultured in standard conditions. On day 7, cDCs were stimulated with CpG (10 *μ*g/mL) or R848 (1 *μ*g/mL). cDCs were harvested 6 hours post stimulation for qRT-PCR analysis. *Pdk1* was normalized to the housekeeping gene *cyclophilin* (a). Standard female unstimulated B6 condition was set to 1 in each experiment. Supernatants were harvested at the 24-hour time point and analyzed using the Griess reaction to measure nitrites (b). Dotted lines at 18 and 40 represent female B6 levels after CpG and R848 stimulation. Mean + SE values are from 3 independent experiments, using one mouse of each strain and sex per experiment (a, b). Two-way ANOVA analysis with Tukey multiple comparisons was used to determine significant activation by CpG or R848 within each group of mice represented by brackets and ^∗^ below the graph. Two-way ANOVA analysis with Tukey multiple comparisons, used to compare differences between B6 and TCSle, is represented by brackets and ^∗^ above the bars. ^∗^ *p* < 0.05, ^∗∗^ *p* < 0.01, and ^∗∗∗^ *p* < 0.001.

Pdk1 (pyruvate dehydrogenase kinase 1) is an inhibitory regulator of the Krebs cycle and controls the amplitude of both the oxidative phosphorylation and fatty acid synthesis. It is downregulated upon activation to increase energy metabolism [60]. We analyzed the expression of *Pdk1* first in standard conditions and found that TCSle cDCs have a decreased expression of *Pdk1* in both sexes, with the baseline levels as low as the levels in B6 cDCs upon activation. TCSle *Pdk1* levels were further decreased upon activation by CpG and R848, suggesting that TCSle cDCs have a higher baseline energy metabolism that can further increase upon activation (Figure 9(a)). This is the first evidence that lupus-prone cDCs have a higher metabolism than wild-type cDCs, and such difference was equally present in female and male cDCs.

Nitric oxide (NO) is synthesized from arginine by inducible nitric oxide synthase (iNOS) [61] and can act as a microbicidal agent [62]. Six to 24 hours after TLR stimulation, cDCs have been shown to produce NO, which participates in sustaining the metabolic shift toward aerobic glycolysis in activated iNOS-expressing cDCs [43]. We measured nitrite as a proxy for NO as reported [63]. Six hours after CpG or R848 stimulation, cDCs did not produce significant levels of NO (data not shown). After 24 hours of stimulation with CpG or R848 in standard conditions, we found high levels of nitrite in the supernatants of all the cDCs, confirming previous reports that TLR stimulation induces NO production in cDCs [43]. TCSle cDCs secreted significantly higher levels of nitrite than B6 cDCs, both from females and males (Figure 9(b)), suggesting a stronger metabolism and commitment to glycolysis in TCSle cDCs. Furthermore, after CpG or R848 stimulation, female cDCs of both strains produced higher levels of nitrite than male cDCs (Figure 9(b): dotted lines at 18 and 40 represent female B6 levels after CpG and R848 stimulation), suggesting a novel sex bias in cDC metabolism.

### 3.10. E2 Enhances the Higher Energy Metabolism of TCSle cDCs

**Figure 10:**
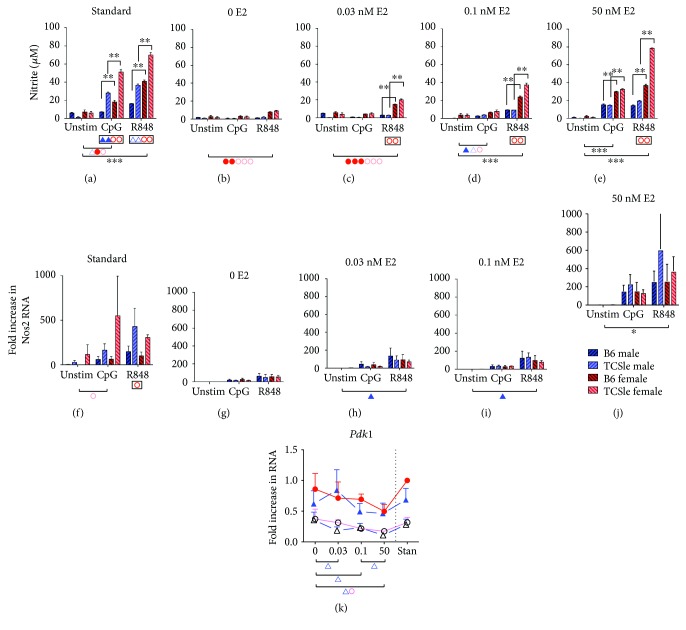
E2 enhances the higher energy metabolism of TCSle cDCs. cDCs from B6 (darker color) or TCSle (lighter color) female (red/orange) and male (blue/azur) mice were cultured in standard conditions or in hormone-depleted conditions supplemented with 0.03 nM, 0.1 nM, or 50 nM E2 (a–k). On day 7, cDC were stimulated with CpG B 1826 (10 *μ*g/mL) or R848 (1 *μ*g/mL). Supernatants were harvested and analyzed using the Griess reaction 24 hours after stimulation (a–e). Six hours post stimulation, cDCs were harvested for qRT-PCR analysis (f–j). *Nos2* (f–j) and *Pdk1* (k) genes were normalized to the housekeeping gene *cyclophilin*. Standard female B6 condition was set to 1. Mean + SE values are from 3 independent experiments, using one mouse per strain per experiment. Two-way ANOVA analysis with Tukey multiple comparisons was used to determine the significant activation by CpG or R848 within each group of mice, represented by brackets below the graph. Black ^∗^ indicates significance within all 4 groups while individual colors/symbols represent significance within a single group (a–k). Two-way ANOVA analysis with Tukey multiple comparisons, used to compare differences between females and males, is represented by brackets and ^∗^ above the graph (a–e). Tukey multiple comparisons, used to compare differences between B6 and TCSle, is represented by a box surrounding red Δ symbol for B6 and TCSle females or a blue O symbol for B6 and TCSle males (a–j). Two-way ANOVA analysis with Tukey multiple comparisons is used to determine the effects of E2 treatment on cDC differentiation within each group of mice represented by brackets and symbols below the graph (j). ^∗^, Δ, and O represent *p* < 0.05. ^∗∗^, ΔΔ, and OO represent *p* < 0.01. ^∗∗∗^ represents *p* < 0.001

Since we have found that E2 can modulate the development and the activation of female and male B6 and TCSle cDCs, we hypothesized that E2 could also modulate the metabolic state of cDCs. The significant differences in nitrite levels linked to activation, sex, and strain (Figures 9(b) and 10(a)) were mostly lost in hormone-depleted conditions, in which cDCs of both strains and sexes secreted very modest amounts of NO upon R848 stimulation and none upon CpGs (Figures 10(a) and 10(b)). With the addition of 0.03 nM E2, the strain bias seen upon R848 stimulation in standard conditions returned, as female TCSle cDCs produced significantly more nitrite than female B6 cDCs (Figure 10(c): significance is shown in the box below the axis). The 0.1 nM E2 dose was sufficient for nitrite production upon both CpG and R848 stimulation, although sex and strain differences were significant only upon R848 stimulation (Figures 10(c) and 10(d)). Finally, 50 nM E2 can recreate the majority of trends seen in standard conditions: a significant increase in the production of nitrite upon CpG and R848 both in female and in male B6 and TCSle cDCs (Figure 10(e)). We observed a significant higher production of nitrite by female than male cDCs in both strains and a significant higher production of nitrite by TCSle cDCs than B6 cDCs. However, the difference between B6 and TCSle cDCs seen in standard conditions (Figures 9(b) and 10(a)) was not rescued upon CpG stimulation (Figures 10(b)–10(e)).

The increase in NO production requires the upregulation of inducible NOS2 expression [61]. Since we have previously found that E2 regulates CXCL10 production at both the transcriptional and translational levels (Figure 7), we determined whether *Nos2* transcript levels followed the trend observed with nitrite levels. cDCs generated in standard culture conditions showed higher expression of *Nos2* by female TCSle cDCs and to a lesser extent by male TCSle cDCs (Figure 10(f)), compared to the levels of *Nos2* expressed by both female and male B6 cDCs. Stimulation with CpG and R848 increased *Nos2* levels, with a higher upregulation in TCSle cDCs than B6 cDCs. cDCs generated in hormone-depleted conditions were unable to support significant increase in *Nos2* levels (Figure 10(g)), while increasing titrations of E2 modestly increased *Nos2* levels in response to CpG and R848 stimulation (Figures 10(h)–10(j)). The 50 nM concentration of E2 could rescue the expression of *Nos2* transcripts to the same level detected in standard conditions, although the differences between B6 and TCSle cDCs were lost. The sex bias towards higher levels of nitrite in female cDCs was not observed with *Nos2* gene expression. As we have seen for the gene expression of ISGs (Figures 5 and 7), the regulation by estrogen is present at the transcriptional and posttranscriptional levels, with strain difference being more affected by the latter.

When we looked at the effects of estrogen on the expression of *Pdk1* transcripts, we found that the hormone depletion did not change the gene expression of *Pdk1*. E2 supplementation decreased the expression in TCSle cDCs in both sexes. Although it did not reach significance, a similar trend was seen with B6 cDCs in both sexes (Figure 10(k)). These results suggest that estrogen can increase the energy metabolism of TCSle cDCs in the absence of other hormones, by decreasing the expression of the inhibitor Pdk1.

In summary, we present the first evidence that lupus cDCs have higher immunometabolism than wild-type cDCs. Specifically, we found decreased levels of the metabolic negative regulator *Pdk1* and increased secretion of NO. While *Pdk1* levels were decreased equally in female and male TCSle cDCs, a sign that both TCSle cDCs equally have increased metabolism of pyruvate [64, 65], NO levels were higher in female than in male cDCs of both strains, suggesting a hierarchy of strength in aerobic glycolysis. TLR stimulation further increased the energy metabolism of both TCSle and B6 cDCs, indicating both a higher baseline and an elevated potential for metabolic activation in TCSle cDCs. Interestingly, nitric oxide production and *Nos2* transcription by cDCs require E2 signaling for cell activation. However, while little differences were seen between sex and strains with *Nos2* transcripts, there is both a sex and strain bias in nitric oxide production, suggesting the ability of E2 to further regulate inducible NOS and the metabolic state at the posttranscriptional level.

## 4. Discussion

Both in SLE patients and in most murine models of lupus, males are less likely to develop disease, but they have higher incidence of renal disorders [66], skin complications [67], and disease severity [68]. Despite this, lupus research has focused predominantly on females due to higher disease prevalence. This gender bias in lupus research does not serve the population of men with SLE well and does not allow us to fully understand the pathogenesis of this complex disease. We have previously reported that cDCs from TCSle female mice show an intrinsic IFN signature that precedes the development of autoimmunity [14]. Here, we show that the IFN signature is equally present in female and male cDCs grown in standard culture conditions and that it is steroidal hormone dependent. We found that E2 enhances the expression not only of the ISGs (Figures 2, 3 and 5) but also of costimulatory molecules (Figure 6), the chemokine CXCL10 (Figure 7), and the proinflammatory cytokines IL-12p70, TNF-*α*, and IL-6 (Figure 8), equally in B6 and TCSle cDCs. When we cultured cDCs with doses of E2 equivalent to what is present in the serum of murine males (0.03 nM) and diestrus female mice (0.1 nM), we found minimal differences between B6 and TCSle cDCs, indicating that TCSle have a normal sensitivity to the effects of estrogen. The finding that the levels of E2 present during human pregnancy (50 nM) were capable of matching or surpassing the ISG, cytokine, and costimulatory molecule expression found in standard conditions suggests that high doses of estrogen can compensate for the absence of other hormones in the medium. Nevertheless, 50 nM E2 was inconsistently capable of yielding differences between B6 and TCSle cDCs, indicating that other components found in standard conditions further mold the TCSle IFN signature. These conclusions were confirmed by the results obtained by adding the SERM/SERD Tamoxifen and Fulvestrant to the standard conditions. Interestingly, these inhibitors reduced *Cxcl10* transcript levels and CXCL10 protein levels to below what was observed in hormone-depleted conditions, hinting at other inhibitory factors in standard conditions that may regulate these responses (Figures 3 and 7). Therefore, we propose that E2 is necessary but not sufficient to the expression of the IFN signature by cDCs and that other factors, especially other steroidal hormones, which are eliminated by the treatment of FBS with charcoal, may contribute to the differences between B6 and TCSle cDCs in standard conditions. Two likely candidates as inhibitors of immune responses are androgens and glucocorticoids, as studies have reported that androgens suppress the activation of key cells of the innate and adaptive immunity [69] and polymorphisms conferring signaling resistance in the androgen receptor inversely correlate with severity of chronic damage [46]. Glucocorticoids have been shown to inhibit DC activation [70] and promote a tolerogenic phenotype [71] although the stimulation through TLR-7 and TLR-9 can confer resistance to glucocorticoids in dendritic cells by promoting NF*κ*B activation in both human cells and murine models of SLE [18]. Moreover, progesterone has been suggested to counteract the effects of estrogens on DC functions in vivo [72]. On the other hand, possible steroidal candidates as immunostimulators are prostaglandins like PGE_2_ that can act as a proinflammatory agent in several models of inflammatory/autoimmune disease, promoting cDC activation and Th17 development [73, 74].

Recently, there was a renewed interest on the heterogeneity of the GM-CSF bone marrow-derived cDCs [57, 75]. Our results add new details to that discussion with the variation of the medium used, IMDM versus RPMI, and the effects of estrogens and steroidal hormones on cDCs so generated. We propose that a rich medium like IMDM promotes the generation of a population of inflammatory cDCs, with a majority of immature or nonactivated CD11^+^ CD11b^+^ and MHC class II intermediate cells and a minority of CD11^+^ CD11b^+^ and MHC class II high cells that spontaneously activated in culture and express higher levels of costimulatory molecules like CD86 (Figure 4) but do not produce yet any cytokines (data not shown). We consider these two populations a gradient of activation of the same cDCs, since MHC class II and costimulatory molecules increase upon TLR stimulation without important changes in the lineage markers, like CD11c, CD11b, and CD115. We do not consider these cell macrophages because they do not express MERTK (data not show). Our results are not really in conflict with those of Helft et al. since they measured *Mertk* RNA and did not show surface protein staining [57], leaving the possibility that even in their protocols, the GM-CSF BMDCs do not express MERTK receptors. No important differences in cDC heterogeneity were measurable between TCSle and B6 cDCs, leading us to conclude that the IFN signature in TCSle cDCs is not due a different cDC composition, but rather to an augmented ability to express ISGs.

Studies in SLE patients indicate dysregulation of costimulatory factors on cDCs such as CD86 [19–21]. Furthermore, inflammatory cytokines such as IL-12 [76], IL-6, and TNF-*α* [77] are dysregulated in SLE. TLR9 and TLR7, respective sensors for CpG and R848, are implicated in SLE [15]. We have previously shown that the response to CpG and R848 stimulation in terms of upregulation of ISGs, CXCL10, and CD86 is type I IFN/STAT-2-dependent, while IL-12, IL-6, and TNF-*α* were regulated in a type I IFN/STAT-2-independent manner [47]. A previous study of pediatric SLE patients reported an IFN signature in both female and males [6] while a recent study of treatment-naïve girls and boys with childhood onset SLE further described a “TNF signature” present in boys but absent in girls [78], suggesting that sex may affect disease differently by promoting type I IFN-dependent and type I IFNs-independent pathways. Recent studies have also shown that prior to diagnosis of SLE, there is elevated type II IFN in the serum of patients [79]. Furthermore, advanced analysis of the IFN signature in adult SLE patients has revealed that there is a modular signature composed of both type I and type II IFN signatures [26]. Collectively, this may explain the clinical efficacy of targeting type I IFN in SLE: while multiple type I IFN-blocking agents in clinical trial are capable of reducing the IFN signature in SLE patients, their effects on the disease are not consistent [80]. We show here that E2 affects both type I IFN-dependent and type I IFN-independent cytokines and costimulatory molecules in both sexes (Figures 6 and 8), indicating that E2 modulates both type I IFN-dependent and type I IFN-independent pathways in both females and males. This suggests that E2 modulation may be beneficial in treating SLE in both sexes.

We found that E2 modulation of cDC activation is different at the transcriptional and posttranscriptional levels, suggesting that E2 affects DC activation through more than one pathway. Indeed, while the highest dose of 50 nM of E2 was required to enhance RNA expression of ISGs, physiologic doses of E2 were sufficient to enhance the production of proteins, either ISGs like CXCL10 or costimulatory molecules and inflammatory cytokines. This pattern of posttranscriptional regulation has been shown to be modulated through effects on the immunometabolism [42].

Recent studies on the intracellular metabolism of DCs have revealed the critical role of the metabolic reprogramming in the response of DCs to environmental changes, from hypoxia to danger signals and cytokines [81]. It was shown that murine wild-type cDCs rapidly increase their glycolytic rate soon after TLR engagement, with a short-term increase in mitochondrial respiration. The upregulation of glycolysis during the early phase of DC activation is essential for NADPH regeneration, fatty acid synthesis, and the enlargement of the endoplasmic reticulum and Golgi. These processes are necessary to produce proinflammatory protein mediators, while the upregulation of RNA transcription seems to require lesser metabolic changes. In iNOS-expressing DCs, such as the murine GM-CSF-derived cDCs, a progressive decrease of the oxidative phosphorylation is induced by the nitric oxide that is produced after the first 8 hours of TLR stimulation. Thus, 24 hours after stimulation, cDCs depend almost exclusively on glycolysis to sustain their function and survival.

Immunogenicity and tolerogenicity of DCs have been proposed to be promoted by anabolic and catabolic processes, respectively. The higher state of activation of TCSle cDCs, which we show here, and of T cells that Wu et al. have reported [45], could be sustained by a higher energy metabolism. Indeed, the result that *Pdk1*, an inhibitory regulator of the Krebs cycle that controls both the oxidative phosphorylation and fatty acid synthesis, is decreased in TCSle cDCs, which is the first evidence that TCSle cDCs have a constitutive higher immunometabolism even in the absence of any exogenous stimulation. The increase in nitric oxide (NO) in TCSle cDCs, which is also used as biomarker of metabolic activation of cDCs [43], indicates that TCSle cDCs respond to activation with a greater shift towards glycolysis. Altogether, these results suggest that, compared to B6 cDCs, TCSle cDCs have a higher baseline mitochondrial respiration and they can generate a stronger aerobic glycolysis upon stimulation with nucleic acids. Since it has been recently suggested that type I IFNs are required for the metabolic shift to glycolysis that occurs during cDC activation [44], we speculate that the IFN signature may contribute to the higher metabolism of TCSle cDCs.

Estrogens have been shown to affect oxidative phosphorylation, glycolytic enzymes, and the glucose uptake upstream of glycolysis in several tissues in the body, especially in the brain [82], while less was known in immune cells. We show here that E2 can further decrease the expression of *Pdk1* in TCSle cDCs and increase the production of nitric oxide, suggesting a novel role for estrogen in increasing the immunometabolism (Figure 10). Moreover, we found that E2 promotes elevated NO production by cDCs in a sex-dependent manner, with female cDCs producing more NO than male cDCs (Figures 9 and 10). This sex bias was even present in cDCs generated with hormone-depleted medium supplemented with physiologic doses of E2, suggesting that the immunometabolism is more affected by E2 than by other hormones present in FBS in a sex-dependent manner.

In conclusion, we show that male TCSle cDCs express the same IFN signature as female TCSle cDCs and respond to the enhancing effects of estrogen, and yet, estrogen alone is not sufficient to recapitulate the difference between TCSle and B6 cDCs. These results suggest that the effect of estrogens on just the IFN signature cannot per se justify the higher incidence of lupus in females, while the effects of estrogens on IFN-dependent and IFN-independent pathways, in conjugation with additional hormonal and environmental factors, are likely necessary for the full development of lupus in genetically susceptible individuals. The results indicating that SERM/SERD inhibit the expression of the IFN signature in cDCs of both sexes *in vitro* warrant that testing the effects of SERM/SERD should be performed in both male and female mice in preclinical studies. Together, the novel data showing a higher immunometabolism in TCSle cDCs, along with the sex bias in NO production and its estrogen dependence, suggest that the immunometabolism may be an important mechanism for the sex bias in lupus pathogenesis and a possible novel therapeutic target in lupus.

## 5. Summary

*β*-Estradiol modulates baseline and TLR-induced activation of lupus conventional dendritic cells; it increases the IFN signature and DC immunometabolism.

## Abbreviations

B6: C57BL/6
cDC: Conventional dendritic cell
DC: Dendritic cells
ER*α*: Estrogen receptor-*α*
E2: 17-beta-estradiol
ISG: Interferon-stimulated gene
IRF: IFN regulatory factor
nM: Nanomolar
NO: Nitric oxide
NOS: Nitric oxide synthase
pDC: Plasmacytoid dendritic cells
Pdk1: Pyruvate dehydrogenase kinase 1
R848: Risiquimod
SERM/SERD: Selective estrogen receptor modulators/degraders
SLE: Systemic lupus erythematosus
TCSle: NZM2410-derived Triple Congenic B6. NZM.*Sle1/Sle2/Sle3.*

## Supplementary Materials

Supplementary Materials Supplemental Figure 1: shows the surface expression of MHC class II and CD115 as markers of differentiation of inflammatory dendritic cells in dendritic cells generated in the presence of different concentrations of estrogens.
