# Supplementary material for: Conventional DCs from Male and Female Lupus-Prone B6.NZM Sle1/Sle2/Sle3 Mice Express an IFN Signature and Have a Higher Immunometabolism That Are Enhanced by Estrogen
Source: J Immunol Res. 2018 Apr 15;2018:1601079. doi: 10.1155/2018/1601079 (PMC5925037; doi:10.1155/2018/1601079)
Supplement: Supplementary Materials — Supplemental Figure 1: shows the surface expression of MHC class II and CD115 as markers of differentiation of inflammatory dendritic cells in dendritic cells generated in the presence of different concentrations of estrogens. [file 1601079.f1.pdf]

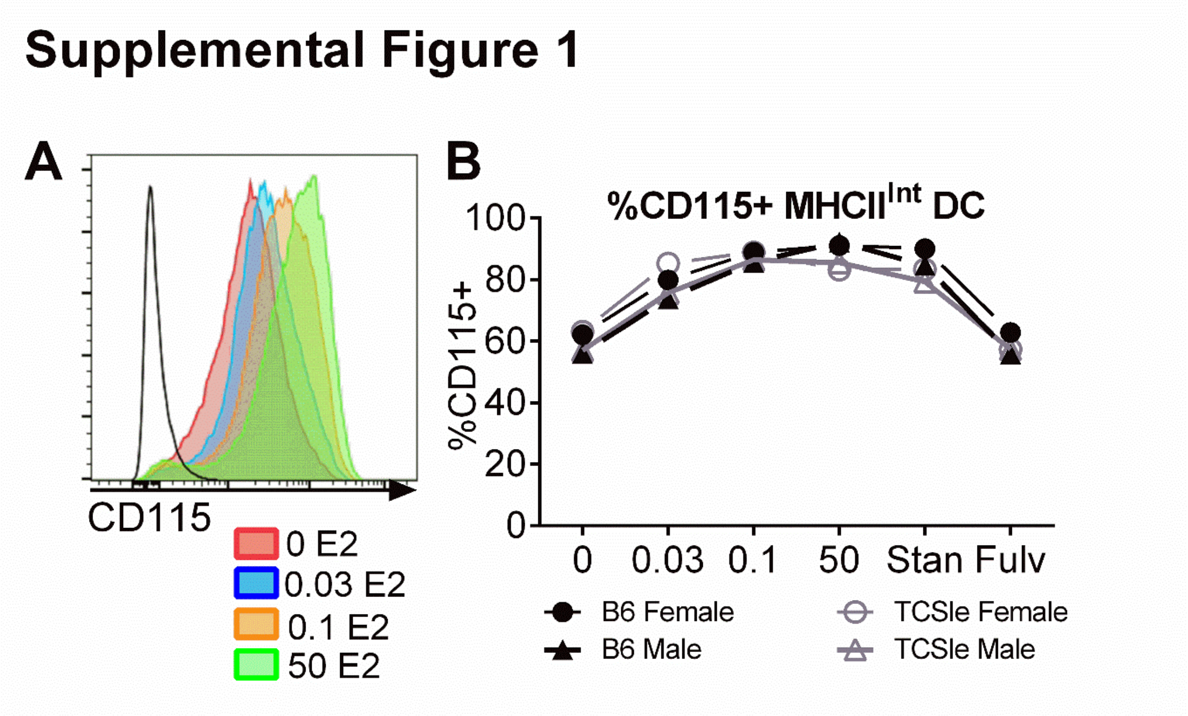

Supplemental Figure 1: *E2 modulates CD115 on B6 and TCSle cDCs* . Bone marrow precursors from B6 or TCSle female and male mice were cultured with GM-CSF in standard phenol red/media conditions or media depleted of phenol red and devoid of steroids (charcoal-treated FBS: 0 E2) and supplemented with 0.03nM, 0.1nM or 50nM 17-β-estradiol (E2). Standard conditions were also supplemented with 1uM Fulvestrant. On day 8, cDCs were harvested and stained with fixable viability dye, and antibodies against CD11c, CD11b, MHCII, and CD115. Samples were gated on singlets, live cells, CD11c^{+}, MHCII^{Int} and CD11b^{+}, then CD115. (**A**) Histogram overlay of CD115 on MHCII^{Int} DC grown in 0 E2 (Red) 0.03nM (Blue), 0.1nM (Orange) or 50nM (Green). Black line is an unstained control. (**B**) %CD115^{+} of MHCII^{Int} DC from B6 (black closed symbols) or TCSle (gray open symbols) female (circle) and male (triangle). N of 1.
